# Characterization and Functional Analysis of a Novel Fungal Immunomodulatory Protein Gene from *Ganoderma leucocontextum* in B16-F10 Mouse Melanoma Cells

**DOI:** 10.3390/ijms26115063

**Published:** 2025-05-24

**Authors:** Jiayi Yang, Mengyuan Jin, Lida Zhang, Yingying Wu, Xuanwei Zhou

**Affiliations:** 1 School of Agriculture and Biology, Engineering Research Center of Therapeutic Antibody (Ministry of Education), Shanghai Jiao Tong University, Shanghai 200240, China; silvan1010@sjtu.edu.cn (J.Y.); jinmengyuan@sjtu.edu.cn (M.J.); zhangld@sjtu.edu.cn (L.Z.); 2 Institute Edible Fungi, Shanghai Academy of Agricultural Sciences, Shanghai 201403, China; wuyingying@sibs.ac.cn

**Keywords:** *Ganoderma leucocontextum*, fungal immunomodulatory proteins, protein expression, B16-F10 melanoma cell

## Abstract

*Ganoderma leucocontextum*, a newly identified species from the Tibetan Plateau, has been mainly studied for its polysaccharides and triterpenoids, with no prior reports on fungal immunomodulatory proteins (FIPs). This study explores the biological activity of FIP-gle2, cloned from *G. leucocontextum* and expressed in *Pichia pastoris*. The effects and mechanisms of recombinant FIP-gle2 (rFIP-gle2) on cell activity and melanin synthesis in mouse melanoma B16-F10 cells were investigated in vitro. The results showed that the *FIP-gle2* gene, with an open reading frame (ORF) of 333 bp, encodes a 111-amino acid polypeptide with a molecular weight of 12.60 kDa and an isoelectric point of 4.48. We achieved a yield of 184.18 mg/L of rFIP-gle2. In vitro functional experiments showed that rFIP-gle2 significantly inhibited the proliferation of B16-F10 melanoma cells and induced apoptosis in a dose-dependent manner, particularly at concentrations above 1 μg/mL. At 3 μg/mL, rFIP-gle2 effectively inhibited tyrosinase activity and reduced melanin content, downregulating microphthalmia-associated transcription factor (*MITF*), tyrosinase (*TYR*), and tyrosinase-related proteins (*TRP-1* and *TRP-2*). Furthermore, RNA-seq analysis indicated that differentially expressed genes in treated cells were enriched in the mitogen-activated protein kinase (MAPK) signaling pathway, with Western blotting confirming enhanced phosphorylation of JNK, ERK, and p38 proteins. Thus, *P. pastoris* is an effective host for rFIP-gle2 production, which shows potential for applications in pharmaceuticals, cosmeceuticals, and food fields.

## 1. Introduction

*Ganoderma leucocontextum*, commonly known as “Tibetan white Ganoderma”, “White meat Ganoderma” or “Tibetan Ganoderma”, is a new member of the Ganodermataceae family that was discovered in 2014 in the Nyingchi region of Tibet [1]. Like other Ganoderma, most of the bioactive components of *G. leucocontextum*, such as polysaccharides [2] and triterpenes [3,4], have been isolated and extensively studied [5,6]. These bioactive compounds are present in higher concentrations compared to other Ganoderma varieties, leading to its recognition as a high-quality Ganoderma species and its rapid emergence as a significant medicinal raw material in the medicinal fungi market [5]. Existing research indicates that the extracts of *G. leucocontextum* exhibit a variety of biological functions similar to those of *Ganoderma lucidum*, including antioxidant [7], antitumor [8,9], neuroprotective [10], and so on. Similarly, current research on *G. leucocontextum* primarily concentrates on the structure and biological activity of its polysaccharides and triterpenes, with only a limited number of studies focusing on its protein components. So far, there has only been research on the isolation and identification of *G. leucocontextum* laccase [11], and no reports have been found regarding fungal immunomodulatory proteins (FIPs). FIPs are a group of small molecules with immunomodulatory activity that have been discovered in fungi. The members of this family have similar structures, with most being homodimers [12]. Presently, more than thirty types of FIPs have been isolated and reported from various edible and medicinal fungi [13,14]. FIPs consist of 106 to 133 amino acid residues, with a molecular weight in the range of 12~15 kDa. Regarding their amino acid composition, FIPs exhibit a notable deficiency in histidine, cysteine, and methionine, while being abundant in aspartic acid and valine.

Existing research indicates that most FIPs possess a wide range of biological activities, such as antitumor, anti-inflammatory, and neuroprotective effects. FIPs can enhance immune function by stimulating T cell activation, proliferation, and cytokine production. Studies have shown that LZ-8 can enhance T cell proliferation and the production of interleukin-2 (IL-2) and interferon-γ (IFN-γ), while inhibiting the production of interleukin-4 (IL-4) [10,15]; FIP-fve also exhibits the ability to activate T cells and enhance IFN-γ release [16]. In addition, FIPs demonstrate the ability to activate macrophages. Li (2019b) found that rFIP-glu can significantly activate RAW264.7 cells [17]; Yu discovered that FIP-hma can significantly upregulate the levels of nitric oxide synthase 2 (NOS_2_), IL-6, interleukin-1β (IL-1β), and TNF-α in macrophages [18]. Furthermore, FIPs can achieve anti-tumor effects by activating the immune responses of host cells and directly acting on tumor cells. Research shows that FIP-nha can negatively regulate the PI3K/Akt signaling pathway, leading to G1/S and G2/M cell cycle arrest by inhibiting Akt phosphorylation. It promotes apoptosis in human lung adenocarcinoma cell line A549 by increasing the expression of Bcl2-associated X (Bax)/B-cell lymphoma-2 (Bcl-2) and cleaved Poly ADP-ribose polymerase (c-PARP). Additionally, it can stimulate autophagy by inhibiting the phosphorylation of the mammalian target of rapamycin (mTOR) [19]. These findings indicate that the effects of FIPs on cancer cells are not singular but result from multiple mechanisms working together to ultimately inhibit cancer cells. Moreover, FIPs also exhibit inhibitory effects on telomerase activity. Liao investigated the effects of purified reFIP-gts on the viability of A549 cells, and the results indicated that reFIP-gts can suppress telomerase activity by inhibiting the expression of human telomerase reverse transcriptase (hTERT) [20]. These various mechanisms enable FIPs to exhibit remarkable effects on tumor cells. For example, rLZ-8 can inhibit the growth of lung cancer cells by positively regulating p53 [21]; rFIP-gap1 demonstrates good hemolytic ability and inhibition effect cancer cell growth [22]; rFIP-gca shows anti-tumor activity against human gastric adenocarcinoma cells (AGS) [23]. Therefore, it can be inferred that FIPs have wide application prospects in the fields of inflammation and tumor related medicine.

This study cloned the fungal immunomodulatory protein gene *FIP-gle2* from *G. leucocontextum* and constructed a *Pichia pastoris* GS115 expression system using the eukaryotic expression vector pPIC9K. An expression system for the recombinant FIP-gle2 (rFIP-gle2) was successfully established, facilitating the in vitro production of rFIP-gle2. The study preliminarily investigated the impact of rFIP-gle2 on the cell viability of mouse melanoma cells (B16-F10) and the activity of tyrosinase, and analyzed its regulatory mechanisms on the signaling pathways associated with melanin synthesis. This study lays the foundation for further in-depth studies and applications of this protein.

## 2. Results

### 2.1. Bioinformatics Analysis of FIP-gle2 Gene

**Figure 1:**
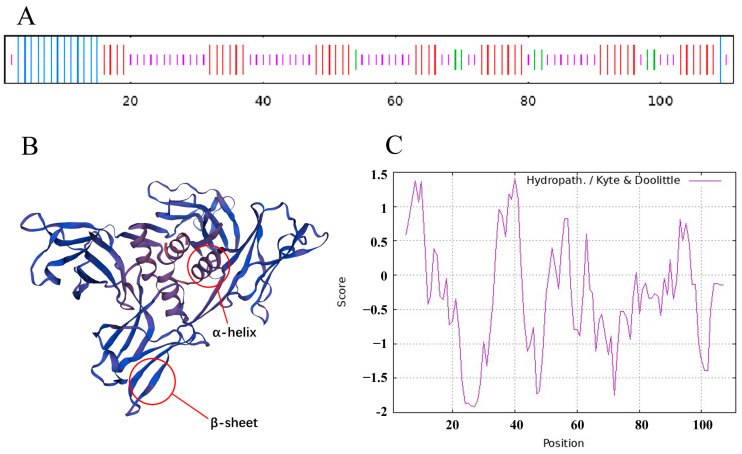
Structural characteristics and physicochemical properties of FIP-gle2. (**A**) Secondary structure prediction of FIP-gle2, where blue represents α-helix, red represents extended strand, green represents β-turn, and purple represents random coil; (**B**) tertiary structure prediction of FIP-gle2; (**C**) hydrophilicity prediction of FIP-gle2.

**Figure 2:**
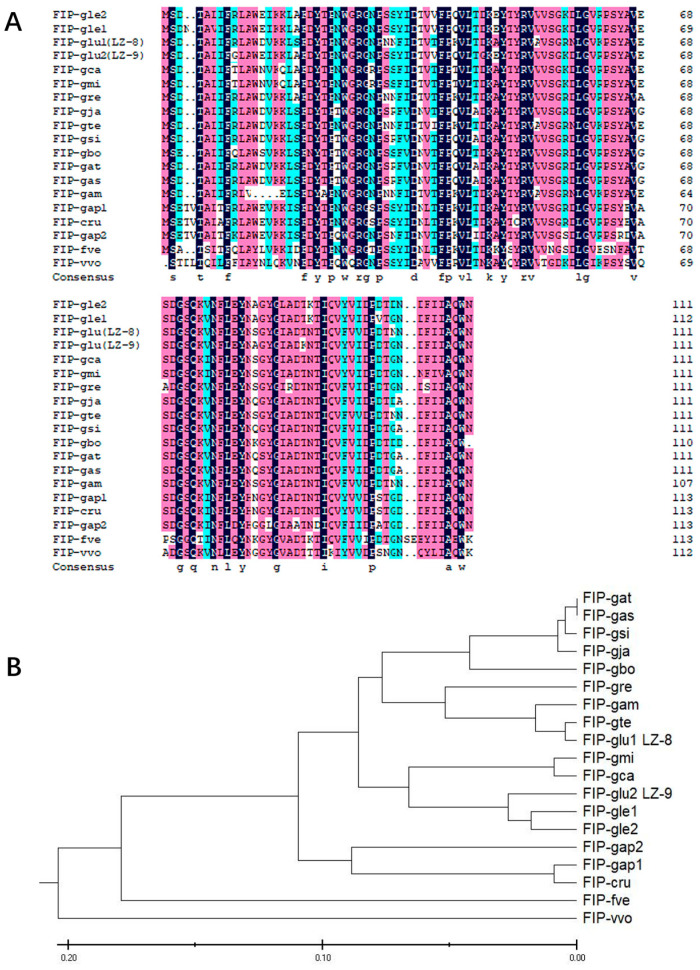
Amino acid sequence alignment and phylogenetic relationship analysis of *FIP-gle2*. (**A**) Amino acid sequence alignment of *FIP-gle2* with selected FIPs, where black indicates completely identical amino acids, and pink and blue represent similar amino acids; (**B**) phylogenetic tree.

In this experiment, a novel *FIP* gene, designated as *FIP-gle2*, was obtained from *G. leucocontextum* using PCR. Sequencing data revealed that the isolated FIP-gle2 gene from *G. leucocontextum* has a length of 333 bp, consisting of 111 amino acids. The molecular weight and the isoelectric point of the protein are 12.60 kDa and 4.48, respectively. By utilizing software for predicting the physicochemical properties of FIP-gle2, it was determined that the secondary structure of FIP-gle2 primarily consists of α-helix, extended strand, β-turn, and random coil, and random coil configurations (Figure 1A). The random coil accounts for the highest proportion, followed by the extended strand and α-helix, whereas the β-turn exhibits the lowest proportion. The outcomes of the tertiary structure prediction for FIP-gle2 are depicted in Figure 1B. The hydrophilicity prediction results for FIP-gle2 are presented in Figure 1C, indicating an overall average hydrophilicity of −0.28, with a minimum value of −1.87 and a maximum value of 1.37, thereby suggesting a predominantly hydrophilic nature. Physicochemical property predictions of the FIP family members are presented in Table 1. The results show that FIP-gle2 is similar to most FIPs in sequence. In terms of amino acid composition, FIP-gle2 is generally deficient in cysteine (Cys) and histidine (His) but rich in valine (Val) and aspartic acid (Asp). FIP-gle2 exhibits high similarity with FIPs isolated and reported from other fungus species. To further elucidate the phylogenetic relationships among FIPs, an amino acid sequence alignment of the FIP-gle2 gene with 18 FIPs isolated from various large fungi species was performed using DNAMAN 6.0 software (Lynnon Corp., Quebec, Canada), and the results are presented in Figure 2A. The similarity between FIP-gle2 and FIP-glu is 82.88%, while the similarity with FIPs from other *Ganoderma species* ranges from 70% to 95%, and the similarity with FIPs from non-Ganoderma species is around 62%. An evolutionary tree was constructed using MEGA11, as shown in Figure 2B. The analysis revealed that FIP-gle2 exhibits a close relationship with LZ-9, FIP-gca, and FIP-gmi, suggesting that these proteins may have diverged from a common ancestral sequence. Furthermore, this finding implies that they may possess similar biological activities. The FIP-gle1 in Figure 2 is the FIP sequence of *G. leucocontextum* obtained from NCBI. Through comparative analysis, it was determined that the FIP-gle2 isolated from *G. leucocontextum* in the present study differs from FIP-gle1, suggesting that it represents a novel protein.

### 2.2. Expression and Purification of rFIP-gle2

**Figure 3:**
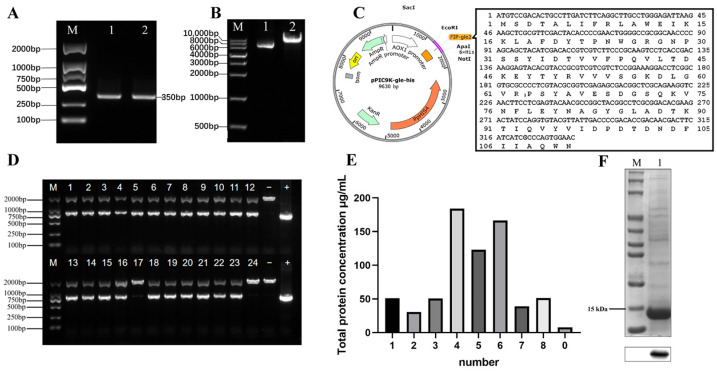
Construction of the recombinant plasmid and identification of yeast transformants. (**A**) Electrophoresis image of the *FIP-gle2* gene amplified by PCR, with lane M representing DNA Marker DL 2000 and lanes 1~2 showing FIP-gle2 fragments; (**B**) electrophoresis image of double digestion of pPIC9K::*X-His*, with lane M representing DNA Marker DL10000 bp, lane 1 showing pPIC9K::*X-His* before digestion, and lane 2 showing pPIC9K::*X-His* after digestion; (**C**) schematic diagram of the recombinant plasmid pPIC9K::*gle2-His*; (**D**) PCR identification of *Pichia pastoris* transformant colonies, with lane M representing DNA Marker DL 2000, lanes 1~24 showing the detection results of yeast transformants containing the plasmid pPIC9K::*gle2-His*, lane − showing the negative control of wild-type GS115, and lane + showing the positive control of the plasmid pPIC9K::*gle2-His*; (**E**) screening of total protein concentration; (**F**) SDS-PAGE and Western blot detection of recombinant FIP-gle2 (rFIP-gle2).

The *FIP-gle*2 gene (Figure 3A) was cloned into the pPIC9K::*X-His* plasmid (Figure 3B) to obtain the recombinant plasmid pPIC9K::*gle2-His* (Figure 3C). The linearized plasmid was electroporated into *P. pastoris* cells, which were then spread onto selective media. The resulting candidate *P. pastoris* transformants were subjected to PCR analysis. The results are shown in Figure 3D, where 22 transformants displayed two bands; the upper band corresponds to the specific fragment in *P. pastoris*, while the lower band corresponds to the specific fragment in the plasmid. This indicates that 22 out of 24 *P. pastoris* transformants contain the recombinant plasmid pPIC9K::*gle2-His*. The selected yeast transformants were fermented in batches for a duration of 96 h. The strain exhibiting the highest total protein concentration was selected as the parental strain for protein induction, achieving an expression level of approximately 184.18 mg/L (Figure 3E). The concentrated yeast suspension of this strain was subjected to SDS-PAGE and Western blot analysis for identification. Given that the target protein is tagged with a 6×His sequence, a mouse anti-6×His monoclonal antibody was selected as the primary antibody for Western blot analysis. The results are shown in Figure 3F, where a specific band was observed near 15 kDa, which is consistent with the expected size of rFIP-gle2.

### 2.3. The Effect of rFIP-gle2 on Cell Viability and Cell Apoptosis

**Figure 4:**
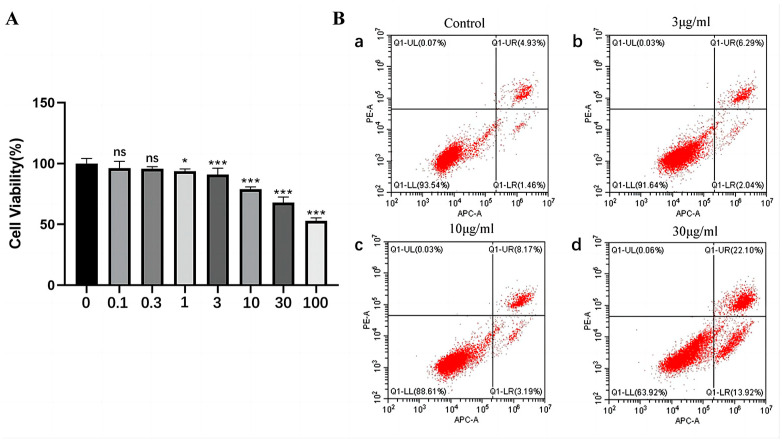
The impact of rFIP-gle2 on the B16-F10 cell state. (**A**) The effects of different concentrations of rFIP-gle2 on the viability of B16-F10 cells, ns for *p* > 0.05; * for *p* ≤ 0.05; *** for *p* ≤ 0.001; (**B**) the effects of different concentrations of rFIP-gle2 on the apoptosis of B16-F10 cells. a–d: apoptosis of B16-F10 cells under different concentrations of rFIP-gle2 treatment.

Before investigating the effect of rFIP-gle2 on melanin synthesis in B16-F10 cells, it is essential to determine its inhibitory effect on cell viability. In a set of seven gradient concentrations (Figure 4A), when the concentration of rFIP-gle2 exceeds 1 μg/mL, the viability of B16-F10 cells exhibits a dose-dependent response to rFIP-gle2, decreasing as the concentration increases. At concentrations of 0.1 and 0.3 μg/mL, there was no significant difference in cell viability, with relative viabilities of 96.20% and 95.78%, respectively. However, when the concentration increased to 1 μg/mL, rFIP-gle2 significantly inhibited the viability of the cell, resulting in a relative viability of 93.7%. As the concentration rose to 3 μg/mL, rFIP-gle2 began to show a highly significant inhibitory effect on B16-F10 cells, with relative viabilities of 90.96% (3 μg/mL), 78.97% (10 μg/mL), 67.91% (30 μg/mL), and 53.00% (100 μg/mL). Concentrations of 3, 10, and 30 μg/mL were chosen for treatment, and the B16-F10 cells were exposed to these concentrations for 24 h. Apoptosis was assessed using a flow cytometer (Figure 4B). The results indicate that as the concentration of rFIP-gle2 increases, the number of viable cells decreases significantly, while the proportion of early and late apoptotic cells increases. Compared to the blank control group, which had a 4.93% early apoptosis rate, the early apoptosis rate increased to 6.29% and 8.17% at concentrations of 3 μg/mL and 10 μg/mL, respectively. When the FIP-gle2 concentration was raised to 30 μg/mL, both the early apoptosis and late apoptosis cell numbers increased significantly. The above experimental results indicate that rFIP-gle2 can promote apoptosis and inhibit activity in B16-F10 cells.

### 2.4. The Effect of rFIP-gle2 on Melanin Synthesis

**Figure 5:**
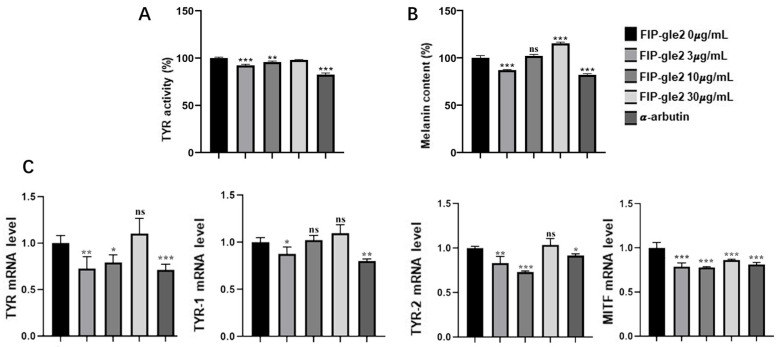
The effect of rFIP-gle2 on important factors in the melanin synthesis process of B16-F10 cells. (**A**) The effect of different concentrations of rFIP-gle2 on the tyrosinase activity of B16-F10 cells; (**B**) the effect of different concentrations of rFIP-gle2 on the melanin content of B16-F10 cells; (**C**) the effect of rFIP-gle2 on the expression of melanin synthesis-related genes in B16-F10 cells. ns for *p* > 0.05; * for *p* ≤ 0.05; ** for *p* ≤ 0.01; *** for *p* ≤ 0.001.

The effects of various concentrations of rFIP-gle2 on tyrosinase activity in B16-F10 cells are shown in Figure 5A. Compared to the control group, the intracellular tyrosinase activity was 92.22%, 95.75%, and 98.08% at concentrations of 3 μg/mL, 10 μg/mL, and 30 μg/mL, respectively. Notably, concentrations of 3 μg/mL and 10 μg/mL exhibited significant inhibitory effects on tyrosinase activity, with the most potent inhibition observed at 3 μg/mL. The effects of different concentrations of rFIP-gle2 on the melanin content in B16-F10 cells are shown in Figure 5B. Compared to the blank group, the intracellular melanin content was 87.08%, 102.07%, and 115.26% at concentrations of 3 μg/mL, 10 μg/mL, and μg/mL, respectively. There was a significant difference between 3 μg/mL and 30 μg/mL, while 10 μg/mL showed no significant difference. Figure 5C illustrates the effect of rFIP-gle2 on the expression of melanogenesis-related genes in B16-F10 cells. Compared to the control group, both 3 μg/mL and 10 μg/mL of rFIP-gle2 significantly inhibited the mRNA levels of *TYR*, with inhibition rates of 28.20% and 20.73%, respectively. The amount of 30 μg/mL of rFIP-gle2 showed no significant regulatory effect on *TYR* mRNA expression. rFIP-gle2 also exhibits regulatory capabilities towards *TRP-1* and *TRP-2*. The results show that rFIP-gle2 at 3 μg/mL significantly inhibited the mRNA levels of *TRP-1*, with an inhibition rate of 12.35%. For *TRP-2*, rFIP-gle2 at 3 μg/mL exhibited an inhibition rate of 16.87%, while at 10 μg/mL, it showed a 26.94% inhibition rate. Compared to the control group, the differences in the inhibitory effects of rFIP-gle2 at three concentrations on the mRNA levels of *MITF* were significant, with inhibition rates of 21.16%, 22.15%, and 13.88%. The above results indicate that 3 μg/mL of rFIP-gle2 has a certain inhibitory effect on important enzymes involved in the melanin synthesis process.

### 2.5. RNA-Seq Analysis

**Figure 6:**
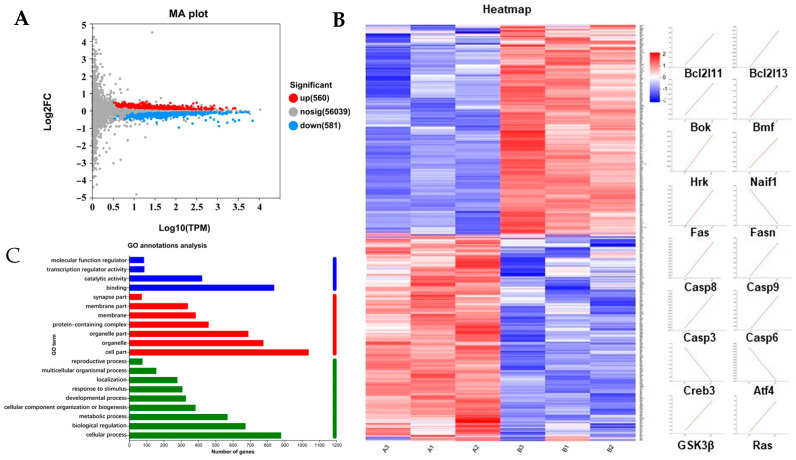
Differential expression genes (DEGs) analysis. (**A**) Expression-level MA differential plot, where the horizontal axis represents the expression values of the differential comparison group and the vertical axis represents the variation in gene expression obtained from the differential expression analysis between the comparison groups. Red points signify genes/transcripts that are significantly up-regulated, blue points signify genes/transcripts that are significantly down-regulated, and gray points represent genes/transcripts that are not significantly different; (**B**) DEGs clustering analysis; (**C**) GO classification statistical chart. The y-axis depicts the secondary classification terms of GO, whereas the x-axis indicates the count of genes/transcripts annotated to each secondary classification. The color coding represents the three primary categories.

**Figure 7:**
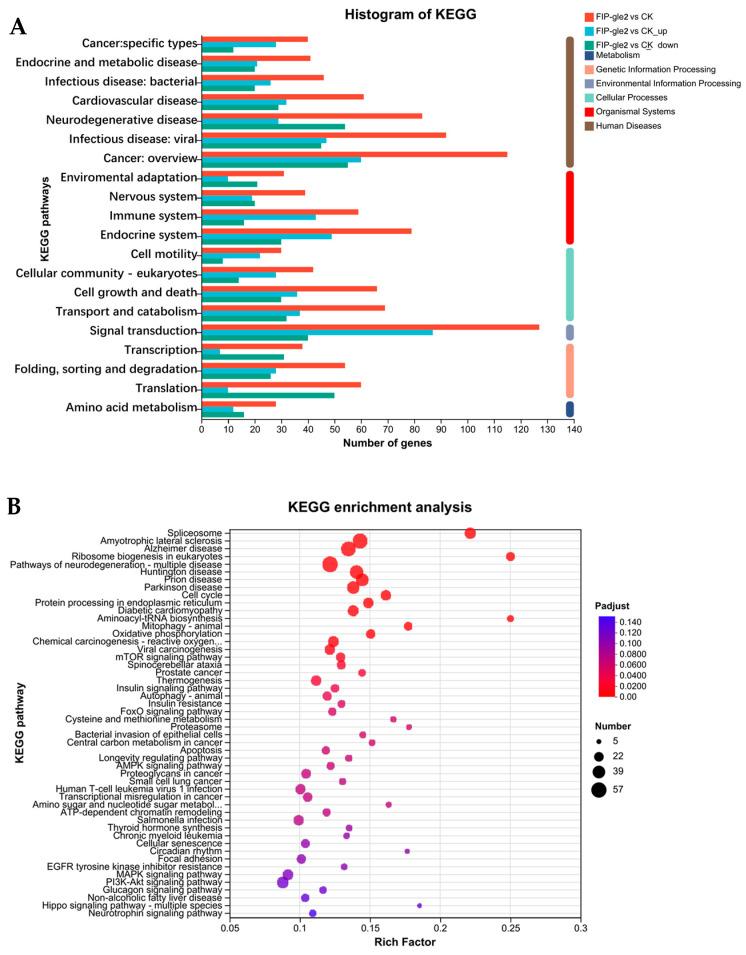
Functional annotation analysis. (**A**) KEGG pathway classification analysis. The y-axis represents the names of KEGG metabolic pathways, while the x-axis represents the number of genes/transcripts annotated to each pathway. (**B**) KEGG enrichment analysis. The y-axis represents the KEGG pathway names, while the x-axis represents the ratio of the number of enriched genes/transcripts to the total annotated genes/transcripts in that pathway. A higher Rich factor indicates a greater degree of enrichment. The size of each point indicates the number of genes within the corresponding KEGG pathway, and the color of the point corresponds to various Padjust ranges.

In order to further investigate the mechanism of action of rFIP-gle2 on melanin synthesis in B16-F10 cells, we conducted transcriptome sequencing analysis on B16-F10 cells treated with 3 μg/mL rFIP-gle2. As shown in Figure 6A, when |log2FC| ≥ 1 and p.adj ≤ 0.05, there were a total of 1141 differentially expressed genes (DEGs) in the group treated with 3 μg/mL rFIP-gle2 compared to the control group, including 560 up-regulated genes and 581 down-regulated genes. Clustering analysis was performed on the DEGs, with results shown in Figure 6B. Treatment with rFIP-gle2 enhanced the expression of genes that inhibit cell proliferation and promote apoptosis in melanoma cells, such as *Bcl2l11*, *Bcl2l13*, *Bok*, *Bmf*, *Hrk*, *Naif1*, *Fas*, *Casp 8*, *Casp 9*, *Casp 3*, *Casp 6*, and *Fasn*. Gene Ontology (GO) is a gene function classification system that encompasses three main aspects: molecular function, cellular component, and biological process. GO analysis of DEGs, as shown in Figure 6C, revealed that in terms of molecular function, binding and catalytic activity were predominant; in terms of the cellular component, the cell part, organelle, and organelle part were predominant; in terms of the biological process, the cellular process, biological regulation, and the metabolic process were predominant. To gain a deeper understanding of the functional classification information of the differentially expressed genes, KEGG pathway classification analysis was performed on the 1141 DEGs (Figure 7A). DEGs were annotated to 20 entries across six biological processes, and the top 50 enriched pathways in KEGG were statistically analyzed (Figure 7B). Signaling pathways related to cancer and cell apoptosis, such as the mTOR pathway, the FoxO signaling pathway, apoptosis, the prostate cancer signaling pathway, and the MAPK pathway, were significantly enriched.

### 2.6. rFIP-gle2 Regulates the MAPK Pathway

**Figure 8:**
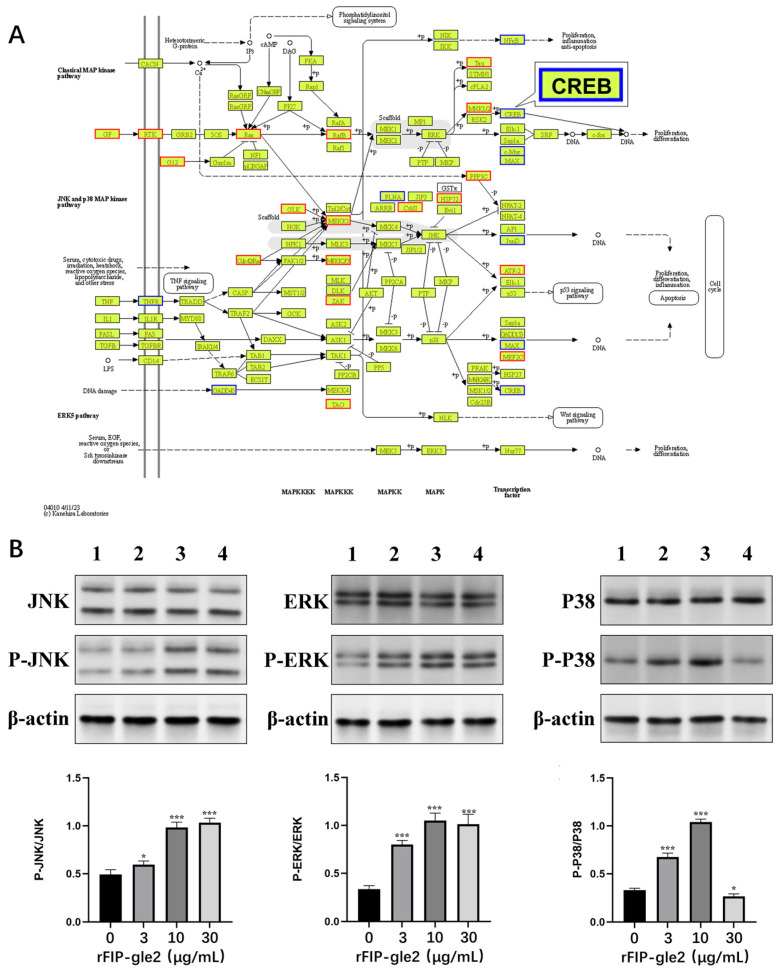
MAPK analysis. (**A**) The effect of rFIP-gle2 on the MAPK pathway is represented, with yellow indicating known genes/known transcripts, red representing upregulated genes, and blue denoting downregulated genes. The figure indicates that CREB gene is downregulated. (**B**) Western blot detection results and phosphorylation levels of MAPK pathway proteins: lane 1, control; lane 2, 3 μg/mL rFIP-gle2; lane 3, 10 μg/mL rFIP-gle2; lane 4, 3 μg/mL rFIP-gle2. ns for *p* > 0.05; * for *p* ≤ 0.05; *** for *p* ≤ 0.001.

Through KEGG enrichment analysis, we found that the differentially expressed genes (DEGs) after treatment with rFIP-gle2 were significantly enriched in the MAPK pathway (Figure 8A), which is involved in apoptosis and melanin synthesis [25]. Therefore, Western blot analysis was conducted to assess the protein translation levels of MAPK signaling pathway components, and the results are shown in Figure 8B. Compared to the control group, the phosphorylation levels of three MAPK pathway proteins were increased in B16-F10 cells treated with 3 μg/mL of rFIP-gle2. The phosphorylation level of JNK was significantly increased, while the phosphorylation levels of ERK and P38 were markedly elevated, suggesting that rFIP-gle2 can induce the phosphorylation of the MAPK pathway.

## 3. Discussion

In recent research, recombinant FIPs have shown various pharmacological activities, such as immune-regulating activity [17,26], antioxidant activity [27], and antitumor activity [14]. As a natural ingredient, FIPs have a clear structure and defined functions, making them easy to produce through biotechnological methods. Therefore, the research and application of FIPs have become a focus of attention for a large number of researchers [5,10].

In this study, a novel *FIP-gle2* gene was cloned from *G. leucocontextum*. Bioinformatics analysis revealed that this FIP shares a high degree of similarity with other FIPs, with a similarity of 82.88% with FIP-glu, and has a similar secondary structure. Therefore, it has been identified as a new member of the FIP family. This study further established a *Pichia pastoris* expression system for rFIP-gle2, with an expression level of approximately 184.18 mg/L. The expression level achieved is comparable to that reported in similar studies conducted in recent years [25,26,27]. Successful expression of rFIP-gle2 in yeast has laid a solid foundation for further research and applications. In this study, the biological activity of rFIP-gle2 was also investigated through in vitro cell experiments. The results demonstrated that rFIP-gle2 can inhibit the activity of B16-F10 cells and induce apoptosis in B16-F10 cells, while low concentrations of rFIP-gle2 treatment can reduce melanin synthesis in B16-F10 cells, suggesting that rFIP-gle2 has a whitening effect to a certain degree.

MAPK represents a group of evolutionarily conserved serine-threonine kinases that can be categorized into four subfamilies: ERK, p38, JNK, and ERK5 [28]. The pathway constitutes a cascade phosphorylation process that plays a crucial role in signaling transduction and regulation within melanoma cell lines. As the most common highly conserved cell regulation pathway in eukaryotes, the MAPK signaling pathway can be activated by different stimuli such as cytokines, hormones, and neurotransmitters, coordinating various biological activities such as gene transcription, protein translation, cell differentiation, and apoptosis [29].

By screening DEGs, we identified several genes associated with apoptosis, including *Bcl2l11*, *Bcl2l13*, *Bok*, *Bmf*, *Hrk*, *Naif1*, *Fas*, *Casp 8*, *Casp 9*, *Casp 3*, *Casp 6*, and *Fasn*. Among these, *Bcl2l11*, *Bcl2l13*, *Bok*, *Bmf*, and *Hrk* are pro-apoptotic proteins within the Bcl2 family [30]. *Naif1* can significantly inhibit the growth rate of tumor cells and induce apoptosis through Casp9 [31]. Fas (factor-related apoptosis) is a commonly recognized death receptor that forms a death-inducing signaling complex upon binding with its corresponding death ligand, activating caspases 8 and 10. Casp 8, Casp 9, Casp 3, and Casp 6 are members of the caspase family responsible for the final execution of apoptosis [32]. The inhibition of Fasn (fatty acid synthase) can promote apoptosis in hepatocellular carcinoma (HCC) cells via the β-catenin/C-myc signaling pathway [33]. Research has shown that activated JNK can activate downstream members of the Casp family through two mechanisms: by antagonizing the anti-apoptotic activity of Bcl2, enhancing the pro-apoptotic activity of Bcl2l 11 through phosphorylation, and stimulating the release of cytochrome C (Cyt-C) [34]. Combining the screening of DEGs, it can be concluded that rFIP-gle2 at low concentrations primarily stimulates endogenous apoptotic pathways in B16-F10 cells by significantly increasing the phosphorylation levels of JNK, leading to the apoptosis of B16-F10 cells.

In addition to participating in basic cellular activities, the MAPK signaling pathway is also involved in the process of melanin synthesis. CREB/MITF is a key pathway in melanin formation [35]. The cAMP response element binding (CREB) protein can be phosphorylated by protein kinase A (PKA), which can enhance the expression of MITF, thereby promoting the expression of downstream melanin-related genes [25]. Research has found that phosphorylation of the JNK/MAPK pathways can also inhibit melanin synthesis. Ro31-8220 (a PKC inhibitor) is a potential inhibitor of the CRTC/CREB-MITF signaling pathway, which can promote phosphorylation of the JNK pathway to inhibit the phosphorylation of CRTC3, thereby inhibiting CREB activity, ultimately affecting MITF expression [36]. Phosphorylation of ERK/MAPK can also inhibit melanin synthesis. Researchers have found that hesperidin (a common antioxidant) can promote MITF degradation by stimulating Erk1/2 phosphorylation, ultimately leading to the inhibition of tyrosinase activity and melanin synthesis [37]. Therefore, the MAPK pathway plays a crucial role in regulating the biological activities of melanoma cells.

Through the screening of DEGs, it was found that rFIP-gle2 at a concentration of 3 μg/mL downregulates the expression of *Creb3* and *Atf4*, while upregulating the expression of *GSK3β* and *Ras*. Both Creb3 and Atf4 belong to the CREB family, and the CREB/MITF pathway is a key pathway in melanin formation [35]. The results of Western blot analysis show that rFIP-gle2 can stimulate the phosphorylation of JNK protein, and JNK can phosphorylate CRTC3 to inhibit CREB-mediated MITF transcription. This suggests that rFIP-gle2 may potentially lower MITF activity downstream by inhibiting the expression of CREB. GSK3β is a key regulatory factor in the Wnt/β-catenin signaling pathway. Inhibition of GSK3β activity leads to the accumulation of β-catenin, which can bind to MITF [38]. GSK3β can also phosphorylate MITF, promoting its degradation [39]. A reduction in GSK3β activity can facilitate the binding of MITF to the promoter of TYR, enhancing melanin production [40]. Ras is a small GTPase that acts as a switch for signal transduction, capable of activating downstream Raf when in the GTP-bound state, thus activating the ERK/MAPK pathway [41]. The activated ERK pathway can promote the degradation of MITF, which is consistent with the results from Western blot analysis. In summary, it can be concluded that rFIP-gle2 at a concentration of 3 μg/mL primarily inhibits the activity of CREB protein and increases the phosphorylation level of ERK protein to promote MITF degradation, thereby suppressing the synthesis of melanin.

## 4. Materials and Methods

### 4.1. Materials

The fruiting bodies of *G. leucocontextum* were provided by Xizang Institute for Food and Drug Control, NMPA Key Laboratory for Quality Control of Traditional Chinese Medicine, Tibetan Medicine. The strains of *G. leucocontextum* were isolated and identified by our laboratory. The strain of *E. coli* DH5α and *Pichia pastoris* GS115, along with the pPIC9K::X-His vector, are preserved in our laboratory. The mouse melanoma cell B16-F10 was provided by the Cell Resource Center, Shanghai Institute for Biological Sciences, and is stored in our laboratory. The seamless Cloning Kit was obtained from Beyotime (Shanghai, China). The Cell Counting Kit (CCK-8), peroxidase AffiniPure Goat Anti-Rabbit lgG (H+L), and the super ECL Detection Reagent were purchased from Yeasen (Shanghai, China). The anti-6x His Tag mouse monoclonal antibody and HRP-conjugated goat anti-mouse IgG were purchased from Sangon (Shanghai, China). The primary antibodies specific for ERK1/2, p-ERK1/2, JNK1/2/3, p-JNK1/2/3, P38, p-P38, and β-Actin were purchased from Abcam (Shanghai, China). Other reagents were of analytical grade.

### 4.2. Cloning and Bioinformatics Analysis of FIP-gle2 Gene

Due to the similarity in protein sequences among the FIPs family members [24], the primers were designed based on *LZ-8* using SnapGene 7.0 (https://www.snapgene.com/) (LZ8F as the forward primer and LZ8R as the Reverse primer (Table 2)). Using the total DNA from *G. leucocontextum* as the material, the gene of interest was prepared through PCR, and the amplification products were sent to Sangon (Shanghai, China) for sequencing. Sequence alignment was performed using DNAMAN 6.0 software (https://www.lynnon.com/). The phylogenetic tree was constructed using MEGA11 software (http://www.megasoftware.net). The similarity was calculated using the Clustalw 2.0.11 (https://www.genome.jp/tools-bin/clustalw). The secondary and tertiary structures of FIP-gle2 were predicted using the NPS online tool (https://npsa-prabi.ibcp.fr /cgi-bin/npsa_automat.pl?page=/NPSA/npsa_sopma.html, accessed on 23 May 2025) and the Swiss-Model online tool (https://swissmodel.expasy.org/, accessed on 23 May 2025), respectively. Hydrophilicity was predicted using the ProtScale online tool (https://web.expasy.org/protscale/, accessed on 23 May 2025). The physicochemical properties were estimated using the Protein Parameter Calc module of TBtools v1.0.

### 4.3. Expression and Purification of FIP-gle2

The expression method for rFIP-gle2 is based on previously published papers from our laboratory [30]. The primer used in this study is shown in Table 2.

### 4.4. Cell Culture

Cell culture methods were based on a previously published paper by Guo et al. [27]. B16-F10 cells were cultured in high-glucose Dulbecco’s Modified Eagle Medium (DMEM) with 10% fetal bovine serum (FBS) (Gibco, NY, USA) and 1% antibiotics (100 U/mL penicillin and 100 mg/mL streptomycin) in a constant temperature incubator at 37 °C with 5% CO_2_. For cell passage, cells were digested using trypsin-EDTA and seeded at a density of 1 × 10^5^ cells/mL.

### 4.5. Cell Viability Assay

The effect of rFIP-gle2 on the viability of B16-F10 cells was assessed using the CCK-8 method [42]. B16-F10 cells were seeded at 100 μL per well in a 96-well plate and cultured for 24 h. Sequentially, rFIP-gle2 was added to the wells at final concentrations of 0.1, 0.3, 1, 3, 10, 30, and 100 μg/mL with PBS as the control. After incubating for 24 h, 10 μL of CCK-8 was added to each well. The optical density (OD) at 450 nm was measured using a microplate reader, and the dosage for subsequent experiments was determined based on the results of cell viability.

### 4.6. Cell Apoptosis Assay

The effect of rFIP-gle2 on the apoptosis of B16-F10 cells was determined using the Annexin V-YS Fluor^TM^ 647/PI Apoptosis Detection Kit (Yeasen, Shanghai, China). B16-F10 cells were seeded at 1 mL per well in a 12-well plate and cultured for 24 h. Sequentially, rFIP-gle2 was added to the wells at final concentrations of 3, 10, and 30 μg/mL. After incubating for 24 h, cells were digested using trypsin without EDTA. After washing the cells twice with PBS, we added 10 μL of PI and 5 μL of Annexin V-YS Fluor^TM^ 647 to each sample and gently mixed them. We incubated them in a dark at room temperature for 15 min, and performed detection within 1 h. Detection was conducted using the APC and PE channels, and double-parameter scatter plots were generated with FLOWJO v10.8 software, where the x-axis represents Annexin V-Alexa Fluor647 and the y-axis represents PI. Each sample run collected data from 10,000 cells.

### 4.7. Determination of Tyrosinase Activity

The effect of rFIP-gle2 on the activity of tyrosinase in B16-F10 cells was assessed using the L-Dopa oxidation method [27]. B16-F10 cells were seeded at 1 mL per well in a 12-well plate and treated with α-MSH for 24 h to induce melanogenesis. Sequentially, rFIP-gle2 was added to the wells at final concentrations of 3, 10, and 30 μg/mL. After incubating for 48 h, the cells were collected and lysed, and the lysate was mixed with a substrate solution containing L-DOPA. The mixture was then incubated at 37 °C for 30 min to 1 h. The optical density (OD) of the samples was measured at a wavelength of 475 nm using a microplate reader.

### 4.8. Determination of Melanin Content

We used the NaOH lysis method to determine the effect of rFIP-gle2 on the melanin content in B16-F10 cells [43]. B16-F10 cells were seeded at 2 mL per well in a 6-well plate and treated with α-MSH for 24 h to induce melanogenesis. Sequentially, rFIP-gle2 was added to the wells at final concentrations of 3, 10, and 30 μg/mL. After 48 h of incubation, we collected and resuspended the cells in 1 mol/L NaOH containing 10% DMSO, and incubated them at 60 °C for 1 h. We measured the OD of the samples at a wavelength of 405 nm.

### 4.9. RT-qPCR Analysis

To investigate the impact of rFIP-gle2 on the expression of genes related to melanin synthesis, real-time quantitative polymerase chain reaction (RT-qPCR) detection was performed on the transcription levels of key genes involved in melanin biosynthesis, including microphthalmia-associated transcription factor (*MITF*), tyrosinase (*TYR*), tyrosinase-related protein-1 (*TRP-1*), and tyrosinase-related protein-2 (*TRP-2*), which regulate the enzymatic reactions leading to melanin production. We designed primers based on the gene sequences of *MITF*, *TYR*, *TRP-1*, *TRP-2* and *GAPDH* (Table 2). Cell RNA was extracted using the RNA Easy Fast Tissue/Cell Kit, and cDNA was synthesized using PrimeScript™ RT Master Mix (Perfect Real Time) (Takara, Beijing, China). The product was stored at −20 °C. *GAPDH* was used as the reference gene to detect the expression levels of *MITF*, *TYR*, *TRP-1*, and *TRP-2*. We calculated the gene expression levels using the 2^−ΔΔCt^ method.

### 4.10. RNA-Seq Analysis

Transcriptome library construction, sequencing, and analysis were all completed by Majorbio Bio-Pharm Technology (Shanghai, China). RNA was extracted from untreated B16-F10 cells and B16-F10 cells treated with 3 μg/mL rFIP-gle2, respectively. The RNA was reverse-transcribed to cDNA after quality assessment and then subjected to sequencing to obtain raw data. The raw sequencing data were filtered using the software fastp (https://github.com/OpenGene/fastp, accessed on 23 May 2025). The quality-controlled clean data (reads) were aligned with the reference genome using the software HiSat2 (http://ccb.jhu.edu/software/hisat2/index.shtml). Quantitative analysis of gene and transcript expression levels was performed using the software RSEM (http://deweylab.github.io/RSEM/). Differential expression analysis was conducted using the software DESeq2 (http://bioconductor.org/packages/ stats/bioc/DESeq2/), with the screening criteria for significantly differentially expressed genes (DEGs) set as follows: FDR < 0.05 and |log2FC| ≥ 1. We utilized the GO database (http://www.geneontology.org/) to categorize genes exhibiting significant alterations in functionality. We used the KEGG database (https://www.genome.jp/kegg/) to classify genes according to the pathways they participate in or the functions they perform, and annotated the DEGs with KEGG pathways. We employed the Python scipy software (https://www.genome.jp/kegg/) to conduct an enrichment analysis of KEGG pathways.

### 4.11. Western Blot

The harvested cells were lysed on ice through the addition of cell lysis buffer, followed by disruption using an ultrasonic disruptor. The protein concentration was determined using a Bradford protein assay kit. The protein was combined with SDS-PAGE (5×) loading buffer and heated at 100 °C for 10 min to ensure complete denaturation. We loaded the samples onto the protein gel and initially performed electrophoresis at 80 V. Once the samples had migrated to the separating gel layer, we increased the voltage to 120 V and continued electrophoresis until completion. We activated the polyvinylidene fluoride (PVDF) membrane in MeOH for 30 s, and then stacked the following layers in order: anode, filter paper, transfer membrane, gel, filter paper, and cathode. After securing the membrane, we placed it in the electrophoresis tank and conducted the transfer at 110 V on ice for 1 h. We placed the transfer membrane in transfer buffer and rinsed it in a shaking incubator for 10 min, repeating this three times. We incubated it in the shaking incubator at room temperature for 2 h for blocking with 5% BSA overnight, and then rinsed it again with transfer buffer. We added the diluted primary antibody into the incubation box, and hybridized it overnight at 4 °C on a shaker. After rinsing with transfer buffer, we added the diluted secondary antibody into the incubation box and hybridized it for 2 h at room temperature on a shaker. After rinsing it again with transfer buffer, we developed it using ECL chemiluminescent reagent and took photographs.

### 4.12. Statistical Analysis

In this study, all data were calculated as the average of at least three repeated experiments, and statistical analysis was performed using the SPSS 13.0 software with one-way ANOVA. Statistical significance was denoted as follows: ns for *p* > 0.05; * for *p* ≤ 0.05; ** for *p* ≤ 0.01; *** for *p* ≤ 0.001.

## 5. Conclusions

In this study, a novel gene (designated as *FIP-gle2*) was isolated from *G. leucocontextum*, and a yeast expression system to produce rFIP-gle2 was successfully developed. Furthermore, the latter’s application potential was assessed. Its bioactivities were confirmed by a series of experiments in vitro. rFIP-gle2 can induce apoptosis in B16-F10 cells by elevating the phosphorylation levels of the JNK pathway, thereby demonstrating a certain degree of anti-tumor activity. Furthermore, rFIP-gle2 may inhibit melanin synthesis by increasing the phosphorylation levels of the JNK and ERK pathways. This study offers a preliminary theoretical foundation for the development and investigation of rFIP-gle2 in the realm of whitening, skincare, and various other cosmetic products. This experiment investigated the effects of rFIP-gle2 on B16-F10 cells. In future studies, healthy adult mice could be chosen as models to explore the whitening effect of rFIP-gle2 through methods such as topical application or injection. Additionally, it may be worthwhile to consider the immunological functions of rFIP-gle2 and investigate its biological activity from the perspective of immune modulation by selecting other cells, such as macrophages.

## Figures and Tables

**Table 1:** Characteristics of FIPs.

FIPs	Homology Compared to FIP-gle2	Resource	Number of Amino Acids (aa)	Molecular Weight (kDa)	Theoretical pI	Gene ID/Ref
FIP-gle2	—	*G. leucocontextum*	111	12.60	4.48	—
FIP-gle1	93.69%	*G. leucocontextum*	112	12.64	4.73	KAI1793471.1
FIP-glu1 (LZ-8)	82.88%	*G. lucidum*	111	13.1	4.84	AAA33350.1
FIP-glu2 (LZ-9)	94.59%	*G. lucidum*	111	12.40	4.55	[24]
FIP-gca	87.39%	*G. capense*	111	12.41	4.44	UOF75531.1
FIP-gmi	85.59%	*G. microsporum*	111	12.40	4.58	3KCW (PDB)
FIP-gre	83.78%	*G. resinaceum*	111	12.46	5.14	AUB29452.1
FIP-gja	83.78%	*G. japonicum*	111	12.48	4.62	AAX98241.1
FIP-gte	82.88%	*G. tenue*	111	12.52	4.84	UOF75530.1
FIP-gbo	82.73%	*G. boninense*	110	12.33	4.62	KT124392.1
FIP-gat	81.98%	*G. atrum*	111	12.45	4.80	AJD79556.1
FIP-gam	78.50%	*G. amboinense*	107	11.98	4.48	[13]
FIP-gap1	77.48%	*G. applanatum*	113	12.74	4.93	AEP68179.1
FIP-cru	76.58%	*C. rutilus*	113	12.65	4.93	AKU37620.1
FIP-gap2	69.37%	*G. applanatum*	113	12.52	4.86	ART88472.1
FIP-fve	62.16%	*F. velutipes*	114	12.73	6.17	ADB24832.1

**Table 2:** The primers used in this study.

Gene	Forward Primer (5′→3′)	Reverse Primer (5′→3′)
*LZ8*	TACGTAGAATTCATGTCCGACACTGCCTTGATCTTCAG	ATGATGGGGCCCGTTCCACTGGGCGATGATGAAGTCG
*Gle*	GGCTGAAGCTTACGTAGAATTCATGTCCGACACTGCCTTGATCTTCAG	TGATGGTGATGGTGGGGCCCGTTCCACTGGGCGATGATGAAGTCG
*AOX1*	GACTGGTTCCAATTGACAAGC	GGC AAATGGCATTCTGACAT
*MITF*	CAAATGGCAAATACGTTACCCG	CTCCCTTTTTATGTTGGGAAGGT
*TYR*	CACCATGCTTTTGTGGACAG	GGCTTCTGGGTAAACTTCCAA
*TRP-1*	CTGTGGATTATTGGGATGA	GTGAGCCACCACTTTGAG
*TRP-2*	GCTGATTAGTCGGAACTCGA	GGTTGGCAGTTTCTCATTATTT
*GAPDH*	TATGTCGTGGAGTCTACTGGT	GAGTTGTCATATTTCTCGTGG

## Data Availability

Data will be made available on request.
